# Perinatal mental health services for women from minority ethnic groups: why patient-centred approach matter

**DOI:** 10.1186/s12916-023-02979-4

**Published:** 2023-10-03

**Authors:** Shanquan Chen, Huyang Zhang, Hannah Kuper

**Affiliations:** 1 International Centre for Evidence in Disability, London School of Hygiene & Tropical Medicine, London, WC1E 7HT UK; 2 Institute for Global Health and Development, Peking University, Beijing, 100086 China; 3 China Center for Health Economic Research, Peking University, Beijing, 100086 China

**Keywords:** Perinatal mental health, Patient-centred approach, Ethnic minorities

## Background

Perinatal mental health refers to the spectrum of psychological conditions that occur during pregnancy (prenatal period) and up to 1 year after delivery (postnatal period) [1]. This includes a range of emotional and behavioural disorders, from mood and anxiety disorders like prenatal or postnatal depression and anxiety to more severe conditions such as postpartum psychosis [1]. One in five women will experience perinatal mental health disorders, which arise from a complex blend of biological (e.g. hormonal changes and altered inflammatory responses), psychological (e.g. stress, insufficient social support, trauma, and negative life events), and sociocultural influences (e.g. socioeconomic status, cultural attitudes towards pregnancy and childrearing, and healthcare access) [2, 3].

Insufficient use of perinatal mental health services can deeply impact women, infants, and families [4–6]. Untreated perinatal mental health issues may result in heightened emotional distress, functional issues, self-harm, and possibly suicide. Infants may face premature birth, low birth weight, developmental delays, and disturbed bonding. Families could experience strained relationships, disrupted dynamics, and the spread of mental health issues. Therefore, adequate perinatal mental health services are essential for the well-being of women, infants, and families.

UK policy stipulates early pregnancy and postpartum mental health screenings, yet a considerable gap between policy and practice exists. Identification rates of mental health issues may fall below 50%, and this disparity is even greater among ethnic minority women [2]. While barriers at the individual, organisational, sociocultural, and structural levels have been identified, specific evidence concerning ethnic minority women remains insufficient.

## Breaking barriers for more accessible and acceptable perinatal mental health services

The recent paper published by Bains et al. explored a pressing concern in perinatal mental health — the provision of appropriate care to women from minority ethnic groups [7]. This study employed a robust qualitative design, employing interviews with twenty-four healthcare professionals, thereby providing rich, diverse, and insightful data that propels us closer to designing more inclusive and effective perinatal mental health services [7]. Three primary themes emerged from the study. The first relates to a pervasive lack of awareness and understanding of perinatal mental illness and service structure, apparent both in healthcare professionals and patients. The second theme brings to light how patients' relationships with their families, friends, and healthcare professionals can act as both facilitators and hindrances to accessing services. The third theme reveals how healthcare professionals promote raising awareness, flexibility, and shared understanding to enhance the accessibility and acceptability of services.

Grounded in the perspectives of healthcare professionals, this study shines a light on the systemic and nuanced barriers that these women face in accessing perinatal mental health services. Undeniably, it underscores the critical need for more patient-centred approaches in this sector. However, as valuable as healthcare professionals' perspectives are, to truly embrace a patient-centred approach, we must include the voices and experiences of the patients themselves.

In defining a patient-centred approach, we typically consider eight key elements: clear communication, continuity of care, involvement in decisions, attention to physical needs, emotional support, involvement of family, effective treatment, and fast access to healthcare advice [8]. These elements are all crucially intertwined, shaping a healthcare experience that is truly responsive to the patient's needs, preferences, and cultural context.

In the context of perinatal mental health services for women from minority ethnic groups, these elements take on added layers of complexity. For instance, clear communication must transcend language barriers and cultural nuances. Emotional support must be sensitive to unique cultural beliefs and biases around mental health. Effective treatment must take into account potentially divergent understandings of mental health between Western medicine and different cultural paradigms. Fast access to healthcare advice must address both systemic barriers and personal hesitations arising from stigma or fear.

Women’s narratives can shed light on how personal relationships can shape their willingness and ability to access services, guiding us to involve families and carers in ways that are more supportive and respectful. Direct engagement with these women can also help to better understand their perspectives on effective treatment and work towards delivering it in a manner that builds trust, ensures continuity of care, and facilitates smooth transitions. Moreover, women’s accounts can elucidate the invisible barriers to clear communication, expose the gaps in emotional support, and identify the factors that may inhibit their involvement in treatment decisions. They can help us comprehend how the fear of stigma, community censure, or the loss of their child might impact their access to perinatal mental health services. Notably, this study's findings regarding the role of peer support workers underline the importance of cultural and linguistic alignment in service provision. Peer support workers, with their lived experiences and cultural closeness, can bridge the gap between health services and the communities they serve, thereby increasing the accessibility and acceptability of these services.

## Conclusions

To summarise, Bains et al.’s study underlines the need for patient-centred approaches in perinatal mental health services for women from minority ethnic groups [7]. As we aim to enhance these services, it is essential to centre the voices and experiences of these women in our strategies. By doing so, we can strive to provide perinatal mental health services that are accessible, effective, empathetic, respectful, and culturally sensitive — embodying the true essence of patient-centred care. More corresponding studies are needed in future.

## Data Availability

Not applicable.
